# Changing computational research. The challenges ahead

**DOI:** 10.1186/1751-0473-7-2

**Published:** 2012-05-28

**Authors:** Cameron Neylon, Jan Aerts, C Titus Brown, Simon J Coles, Les Hatton, Daniel Lemire, K Jarrod Millman, Peter Murray-Rust, Fernando Perez, Neil Saunders, Nigam Shah, Arfon Smith, Gaël Varoquaux, Egon Willighagen

**Affiliations:** 1 Science and Technology Facilities Council, Didcot, Harwell Oxford, UK; 2 Public Library of Science, San Francisco, USA; 3 Leuven University, Heverlee, Belgium; 4 Michigan State University, East Lansing, MI, USA; 5 University of Southampton, Southampton, UK; 6 Oakwood Computing Associates Ltd, Surrey, UK; 7 LICEF Research Centre, Montreal, QC, Canada; 8 University of California, Berkeley, USA; 9 University of Cambridge, Cambridge, UK; 10 CSIRO, Sydney, Australia; 11 National Center for Biomedical Ontology, Stanford, USA; 12 Adler Planetarium, Chicago, USA; 13 INRIA, Saclay, France; 14 Maastricht University, Maastricht, Netherlands

## 

The past year has been an interesting one for those interested in reproducible research. There have been great examples of replicability [1,2] in research communication, and examples of horrifying failure of reproducibility (as described in [3]) with serious questions being raised on the ability of our current system of research communication to guarantee, or even encourage, that published research be reproducible or replicable.

When we launched the call for papers for Open Research Computation in late 2010 we saw a clear need for higher standards. Computational research should stand out as an exemplar of just how reproducible research can be, yet it falls short more often than not. With modern computational tools it is entirely possible to provide packages which allow direct replication of results. It is possible to provide data and code in the form of a functional virtual machine image along with automated tests to ensure everything is working as expected. But alongside this we can support the reader’s ability to modify and re-purpose tools, to run them against new data, indeed to support efforts to deliberately break the system to identify its limitations. In short, to do what we are supposed to do as scientists – replicate, reproduce, and test the limits of our models and understanding.

We deliberately set the bar high, because we felt it should be high, and because we felt that current standards were, in general, not high enough. Over the past year commentaries [4-6] have supported these principles, recognizing that there are serious problems – but few have actually backed up those words with actions. As with data, so with code, journal statements requiring that it be available often lack substance – how is it to be made available – and policies generally lack teeth.

As we looked at papers for ORC we set higher standards. We demanded that testing of the code be described. We required that we be able to fork the relevant code before formal acceptance of the paper. We looked hard at the documentation. Are the papers and the code being published today perfect? No. Are they an improvement over the average? Absolutely. Indeed a big part of that improvement is in ensuring that those imperfections can be identified, and worked on for the future.

But arguably we may have set the bar too high. Creating code and documentation to the level that we wanted to see is hard work. And the bottom line is that relatively few groups or projects are willing to put in that work, particularly for publication in an, as yet unproven, journal. So we struggled to get submissions at the level we wanted. And here we ran into the second problem.

In approaching our goals in the form of a journal we made an implicit compromise. We wanted to do something radical, but we did it in a form that was familiar and conventional. This was a deliberate tactical move. But problems arise from the straightjacket that a conventional journal form creates. The first is the issue of indexing. To get indexed requires a journal to publish in each month of a calendar year. If you’re trying something new this isn’t so straightforward.

In the end we have decided to fold ORC into another BMC journal, Source Code in Biology and Medicine, as a thematic series. This has a lot of advantages. It means there is less pressure to immediately get the submission numbers up, making it possible to take a longer term approach, and adapt over time as interest, demand and standards change. We can pull papers in from across the BMC portfolio and offer the “badge” of ORC certification as an extra bonus. In this way we get most of the advantages of a journal but avoid many of the pitfalls. But part of our strategy was the thinking that the journal could have a high Journal Impact Factor (JIF) if a reasonable proportion of papers, being highly used tools, got significant numbers of citations. But you only get JIF’s for individual journals – not for collections of papers. And re-publishing articles that have “already been published” (whatever *that* means in a web based world) is a definite no-no.

Many of us have felt for a long time that the construct of the journal places artificial constrictions on what we can do in research communication. The restrictions placed on what counts as a journal, what is allowed in a journal, by indexing services are a real drag on innovation. This raises the question of what is required to place innovation at the heart of the system we use to communicate research. How can we build the systems and infrastructure that we use in a way that actively encourages innovation?

In part, the answer lies with the papers that we are publishing today, and that we will continue to review for acceptance into this thematic series. Show our working, use open tools, enable others to replicate and to fork our work and our tools and our systems. Embed within those systems the measures of reputation and use and re-use that support the most successful open source projects and arguably also the research projects that are most successful at using the web as a resource.

Raising the standards of computational research is an important task, and one that we will continue to pursue by identifying and celebrating papers describing code that reaches those high standards that we have set. But equally we hope to keep learning from the process about what we can apply both to research more generally beyond pure computation and to the process of communicating that research.

We need more than just reproducible computational research, we desperately need a step change in our expectations and in the incentives for communicating research in a reproducible form more generally. We need educators and the materials to support them in raising awareness and experience. And we need the development of policy and standards that help us move towards a world where reproducibility and replicability are minimum standards not aspirations. ORC will not be doing this as a separate journal, but through the thematic series we will continue to promote the principles and the lessons we can learn. The problems are real and we need to tackle them.
